# Single-center, prospective study evaluating safety and efficacy of a new endoscopic hemostat system in non-variceal upper gastrointestinal bleeding

**DOI:** 10.1055/a-2650-2692

**Published:** 2025-07-29

**Authors:** Hardik Rughwani, Rajat Garg, Mohammed Faisal Habeeb, Nitin Jagtap, Zaheer Nabi, Pradev Inavolu, Shreevyshnavi Aachi, Mohan Ramchandani, Darisetty Santosh, Gauri Nayak, Sundeep Lakhtakia, Nageshwar Reddy

**Affiliations:** 1 78470 Dept. of Medical Gastroenterology, Asian Institute of Gastroenterology (AIG Hospitals), Hyderabad, India; 2 2569 Dept. of Gastroenterology and Hepatology, Cleveland Clinic Foundation, Cleveland, United States; 3 1442 Dept. of Medicine, NHS Ayrshire and Arran, Ayr, United Kingdom of Great Britain and Northern Ireland; 4 78470 Dept. of Anaesthesia, Asian Institute of Gastroenterology (AIG Hospitals), Hyderabad, India; 5 29566 Dept. of Medical Gastroenterology, TNMC & BYL Nair Ch. Hospital, Mumbai, India

**Keywords:** Endoscopy Upper GI Tract, Non-variceal bleeding, Ulcers (peptic and other), Endoscopy Small Bowel, Small intestinal bleeding

## Abstract

**Background and study aims:**

Endoscopic spray therapy has been shown to be effective and safe in managing upper gastrointestinal bleeding (UGIB). We aimed to evaluate safety and efficacy of the novel powder-based Resolv Endoscopic Hemostat System in managing UGIB.

**Patients and methods:**

This was a single-center, prospective, single-arm study conducted from July 2022 to February 2023. It aimed to evaluate safety and efficacy of a novel plant-based polysaccharide, the Resolv Endoscopic Hemostat System, in achieving hemostasis in adult patients diagnosed with non-variceal upper gastrointestinal bleeding (UGIB) (Forest 1b/oozing bleeding). Participants in this study underwent endoscopy and received monotherapy treatment using the Resolv Endoscopic Hemostat System. Outcomes of interest were adverse events (AEs) related to the device within 72 hours and 30 days, immediate hemostasis, and rebleeding rates within 72 hours of the index procedure.

**Results:**

A total of 59 patients (71.2% men) with mean age of 55.3 ± 14.2 years were included in the study. Causes of bleeding included post-polypectomy (n = 35, 59.3%), gastric ulcers (n = 13, 22%), malignant tumor (n = 4, 6.8%), post-biopsy-related needing hemostasis (n = 3, 5.1%), congestive gastropathy (n = 2, 3.4%), duodenal ulcer (n = 1, 1.7%), and portal hypertensive duodenopathy-related (n = 1, 1.7%). Resolv achieved a 100% success rate for immediate hemostasis with a 72-hour rebleeding rate of 5.1%. There were no AEs related to the device or mortality.

**Conclusions:**

Resolv Endoscopic Hemostat System is a safe and effective device for achieving immediate hemostasis in patients with non-variceal upper gastrointestinal bleeding. Future studies are required to examine its widespread adoption and applicability.

## Introduction

Upper gastrointestinal bleeding (UGIB) is a common clinical problem with a reported incidence of 47/100,000 with a 30-day mortality of up to 10% 1 2 . Endoscopic therapies performed within 24 hours after onset of gastrointestinal bleeding are effective in controlling the bleeding and need for surgery 1 3 . Although there have been advances in endoscopic hemostasis, there has also been increased use of novel anticoagulants and antiplatelet agents, increasing risk of bleeding. Also, endoscopic interventions such as endoscopic mucosal resection (EMR) and endoscopic submucosal dissection (ESD) have increased rates of post-procedure bleeding 1 . Advances in endoscopic hemostasis have occurred, from conventional injection of dilute epinephrine to applying thermal energy and more recently, mechanical hemostasis 2 4 5 .

Thermal therapy and hemoclips are cornerstones of treatment of gastrointestinal bleeding 5 . Endoscopic spray therapies are increasingly used to provide hemostasis in difficult-to-access bleeding sites, inadequately controlled bleeding from standard therapies, or diffuse bleeding from a tumor or endoscopic intervention site 4 6 . Hemostatic powders (HPs) offer an alternative approach to managing gastrointestinal bleeding. Their functionality encompasses two distinct mechanisms: first, they serve as a mechanical barrier to prevent active bleeding, and second, they prompt anticoagulant effects via systemic absorption. On contact with the bleeding site, these powders swiftly create a barricade on the vascular wall, halting the bleeding. Subsequently, as the water in the blood gets absorbed, it elevates the concentration of coagulation factors, thereby enhancing clot formation. This dual-action approach contributes to their effectiveness in addressing GIB 6 . Spray-based hemostasis is now used as a primary or adjunct therapy. A recent randomized trial comparing a polysaccharide-based endoscopic spray therapy with conventional therapy showed equal efficacy of the spray agent for immediate hemostasis, rebleeding, and 30-day mortality 7 .

In this study, we aimed to investigate safety and efficacy of the novel plant-based powder Resolv (Hemostasis LLC, Minnesota, United States) Endoscopic Hemostat System for treating UGIB.

## Patients and methods

### Study design

This was a single-center, prospective, single-arm study to evaluate the safety and efficacy of the Resolv Endoscopic Hemostat System in achieving hemostasis as monotherapy in adult subjects diagnosed with acute non-variceal UGIB (NVUGIB) (Forrest 1b/oozing bleeding) at a tertiary hospital in India.

### Population (inclusion and exclusion criteria)

Inclusion criteria were adult patients (aged 18–90 years) with confirmed NVUGIB of Forrest Classification 1b who could be followed up for at least 72 hours post-operatively and then at 30 days. Informed consent was obtained from the patients before enrolment. Patients with hemodynamic instability (blood pressure < 90/60 mm Hg and/or pulse > 110/bpm) at time of endoscopy, uncorrected coagulopathy, peptic ulcers previously treated with other modalities less than a week before the study, presence of a vascular shunt, known hypersensitivity to potato starch, known case of decompensated chronic liver disease, pregnant or lactating patients, patients with contraindications to endoscopy, and unable to interrupt anticoagulants and antiplatelet agents for at least 72 hours after endoscopy were excluded.

### Intervention (pre-procedure, procedure, post-procedure)

After screening and meeting inclusion criteria, patients were enrolled in the study. After enrolment, each patient underwent detailed screening, including a review of history and medications, thorough physical examination, and routine lab tests from the research team. The Glasgow-Blatchford scores were also obtained after enrollment.

Before endoscopy, patients were resuscitated hemodynamically, and any underlying coagulopathy was corrected. All patients underwent upper gastrointestinal endoscopy within 24 hours of hospital admission or within 24 hours of clinical onset of GIB for inpatients. Each patient received up to two cartridges of Resolv Endoscopic Hemostat powder during the index procedure, not exceeding 33.5 g. Resolv hemostatic powder was used as monotherapy in all included patients. Resolv hemostatic powder spray can be delivered continuously or intermittently until hemostasis is confirmed. Once the bleeding was controlled (first application), the area was observed for 5 minutes, and if during that time there was a recurrence of bleeding, a second application was made, and the site again was observed for 5 minutes. If rebleeding occurred after the second application, the endoscopist proceeded with the standard of care as appropriate (injection, cautery, mechanical device, embolization, or surgery). For bleeding from peptic ulcer disease, intravenous omeprazole infusion (80 mg bolus followed by 8 mg/hr for 72 hours as appropriate) was also administered as standard of care.

**Fig. 1:**
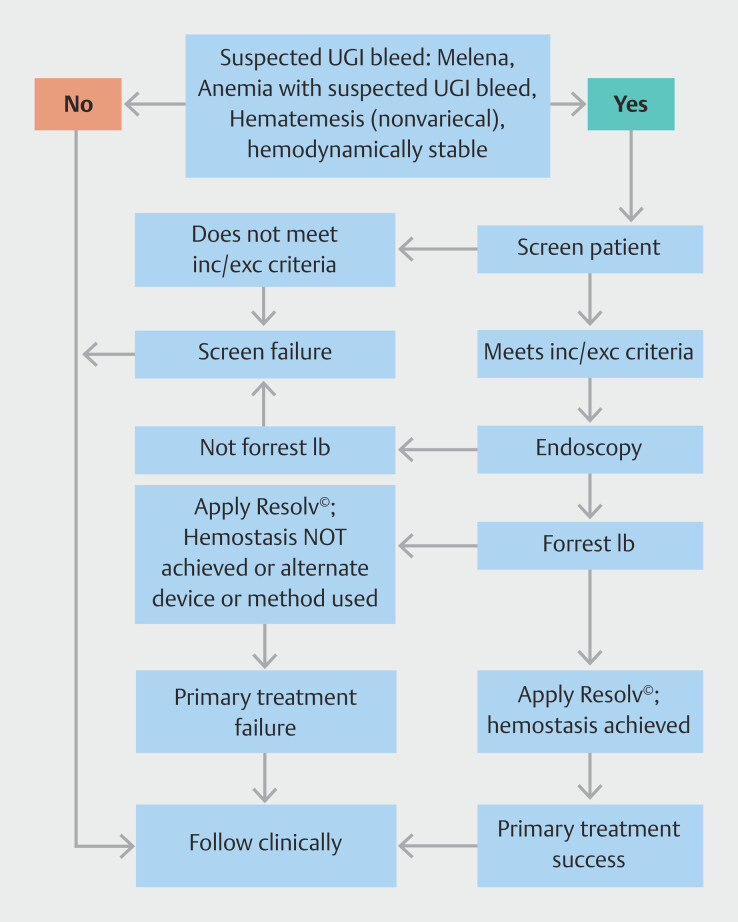
Schematic diagram of assessment and management of UGI bleeding. inc/exc: inclusion/exclusion.

Post-procedure, patients were kept nothing per mouth for 24 hours and monitored for 72 hours for rebleeding (symptoms of hematemesis, hematochezia, melena, or hemodynamic instability). In case of rebleeding, the degree of hemoglobin drop and the number of blood transfusion units required were noted, and a repeat endoscopy was performed to assess the treated site. Serum glucose was measured 2 and 4 hours post-procedure (via finger prick/blood sample collection). Blood glucose was monitored due to active content of Resolv Hemostat powder because it is composed of plant-based starch polysaccharide. Patients were followed up at 30 days post-procedure (in person or by telephone) to monitor occurrence of any serious adverse events (SAEs) and mortality. A schematic of endoscopy, treatment, and assessment of hemostasis is shown in Fig. 1 .

### Device description and functionality

**Fig. 2:**
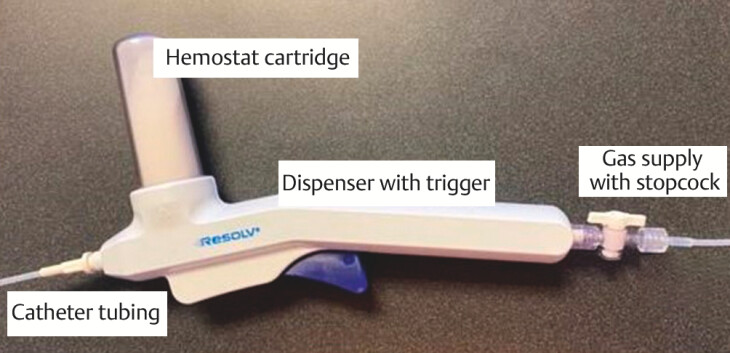
Resolv Hemostat device components.

**Fig. 3:**
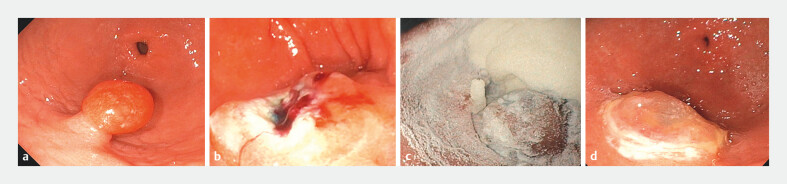
**a** Gastric polyp pre-resection. **b** Post-polypectomy bleeding. **c** Immediate application of the Resolv hemostatic powder. **d** Endoscopic image showing a protective layer of the Resolv powder on the bleeding site.

The Resolv Endoscopic Hemostat System is a sterile prescription device intended for use under the care of a healthcare professional for hemostasis of non-variceal GIB. The device consists of a hemostat powder and dispenser components provided in a tray with a Tyvek lid acting as the sterile barrier. The hemostat powder is in a capped cartridge and packaged in a foil pouch. The dispenser components include a flow dispenser, two catheter tubing sets and a gas source tubing set. The gas source tubing set is connected to a medical CO₂ gas source that is pressure- and flow-controlled. The pressure is set to 10 psig maximum and the flow is set within the range of 0.6 to 1.0 L/min. Before use, the device is assembled by attaching the tubing sets and hemostat cartridge to the flow dispenser. For treatment of bleeding sites in the gastrointestinal tract, with CO₂ flow initiated, the catheter tubing is fed down the working channel (≥ 2.8 mm diameter) of an endoscope until the catheter tubing end is seen beyond the distal end of the endoscope. The tip of the catheter tubing is held at least 1 to 2 cm away from the bleeding tissue to be treated and the hemostat powder is delivered to the site by depressing the trigger on the dispenser handle. The endoscope distal end controls aim at the flow and placement of the hemostat powder. The trigger is released to stop the flow of powder. Fig. 2 shows the components of the device and Fig. 3 shows the pre- and post-procedure application images.

The hemostat powder is identical to the commercially available topical hemostat NexStat Plus, composed of plant-based polysaccharides, which received 510(k) clearance with K122886 as a topical wound dressing. It has been extensively tested in animal models and has shown excellent efficacy and safety profiles, but it has not been studied in humans for UGIB. The hemostatic particles quickly dehydrate the blood cells, resulting in hemoconcentration of platelets, serum proteins, and fibrinogen, leading to clotting that controls bleeding. Concentration of serum proteins and cells produces a viscous gel. Normal platelet activation and fibrin deposition within the congealed blood produces a clot that limits further bleeding. Potential benefits of the Resolv Endoscopic Hemostat System include ease of use, accessibility to bleeding sites in difficult gastrointestinal locations, and ability to treat a wider bleeding area. The hemostat powder is composed of a plant-based polysaccharide without animal proteins or unique mixing or storage requirements.

### Outcomes and definitions

The primary objective was to evaluate the safety of the Resolv Endoscopic Hemostat System. Primary endpoints were rates of AEs/ SAEs within 72 hours after hemostat application, rates of device-related SAEs within 30 days of procedure, and all-cause mortality rates within 30 days of index procedure (all-cause).

The secondary objective was to evaluate the efficacy of the Resolv Endoscopic Hemostat System in controlling non-variceal GIBs (Forrest Ib). Secondary endpoints were the rate of immediate hemostasis on index endoscopy, rebleeding rates within 72 hours of index procedure/application, and need for surgery or an alternative hemostatic method to achieve hemostasis within 72 hours in case of failure of the hemostatic system.

### Ethical considerations

This study was registered at the Clinical Trials Registry India with registration no. CTRI/2021/12/038478. The device manufacturer, Hemostasis LLC, provided funding for the study. Our Institutional review board approved the study. Informed consent was obtained from patients or their legal representatives.

### Statistical analysis

Study population baseline demographics and outcomes of interest were collected. Categorical variables were expressed as percentages and continuous variables as medians (interquartile ranges) or means with standard deviations. Before the study, an estimation of the number of cases was performed. A literature review, performed before the study, supported use of a 9% margin in two-sided 95% confidence interval calculations. Therefore, it was determined with a power of 90% that a sample size of 48 patients was required to test whether the AE rate within 30 days of the index procedure was ≤ 5%. For this study, the N was increased to 60 to provide for some dropout in the study. No formal hypothesis testing was performed for this study. Descriptive statistics were calculated with Microsoft Excel (Microsoft, California, United States).

## Results

### Baseline characteristics

Video showing bleeding Forrest 1b ulcer, assembly and application of Resolv endoscopic system on actively bleeding ulcer.Video 1

**Fig. 4:**
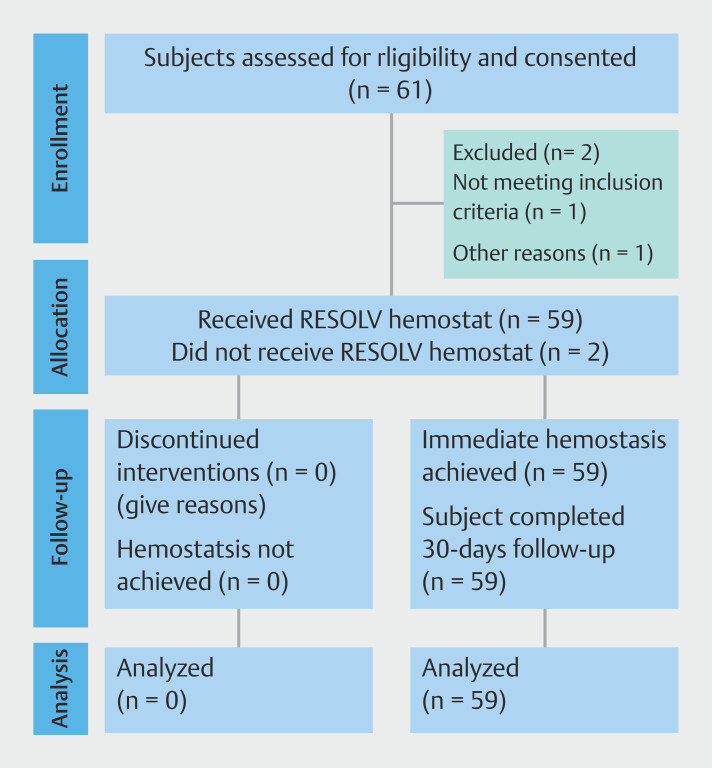
Flowchart showing patient selection through the study.

Of the 61 patients considered for the study, two were excluded due to progression of bleeding severity from Ib to Ia. A flowchart of patient selection is shown in Fig. 4 . A cohort of 59 individuals, 42 men and 17 women with an average age of 55.3 ± 14.2 years, underwent exclusive treatment using the Resolv Hemostat System for NVUGIB. Detailed information regarding baseline patient characteristics and the rationale for implementing the Resolv system can be found in Table 1 . Most patient required one hemostat cartridge (n = 55, 93.22%) and only 6.7% (n = 4) required two hemostat cartridges. Application of the Resolv Hemostat System for Forrest 1b ulcer for treatment is shown in Video 1 .

**Table:** **Table 1** Baseline characteristics of the included population.

Factor	Value*
Age (years)	55.3 ± 14.2
Male	42 (71.2%)
Female	17 (28.8%)
Diabetes	17 (29.3%)
HTN	21 (35.6%)
Compensated CLD	7 (11.8%)
Anticoagulant use	0
Prothrombin time (seconds)	13.5 ± 3.3
Partial thromboplastin time (seconds)	31.5 ± 4.3
Blood urea	37.0 ± 29.3
Platelets	235.6 ± 106.7
Baseline serum glucose	122.8 ± 50.1
Glasgow-Blatchford score	4.4 ± 3.8
Bleeding site	
Gastric	52 (88.1%)
Duodenum	4 (6.8%)
Gastroesophageal junction	3(5.1%)
*Values are shown as n (%) or mean ± standard deviation. CLD, chronic liver disease; HTN, hypertension.	

The most common cause of UGIB was post-gastric polypectomy bleeding in 35 cases (59.3%), followed by gastric ulcer bleeding in 13 cases (22%), malignant tumor bleeding in four cases (6.8%), post-biopsy-related bleed in three cases (5.1%), congestive gastropathy-related bleed in two cases (3.4%), duodenal ulcer bleeding in one case (1.7%), and portal hypertensive duodenopathy-related bleed in one case (1.7%). Resolv served as the exclusive monotherapy approach for all included patients.

### Clinical outcomes

Regarding primary outcomes, there were no AEs or SAEs related to the device within 72 hours of its use and all-cause 30-day mortality was 0% ( Table 2 ). No procedure-related AEs related to device application, such as intestinal obstruction or perforation, occurred. The rate of primary/immediate hemostasis achieved through Resolv monotherapy was 100% in all 59 patients during the initial procedure. The post-procedure rebleeding rate was noted to be 5.1% (n = 3). Among these patients, two required repeat endoscopy for hemostasis, with one needing endoscopic clipping and the second a repeat application of Resolv powder. The third patient showed clinical signs of rebleeding with melena, which resolved with conservative treatment without requiring a second endoscopy. The catheter clogging rate for the Resolv system was 3.4 % (n = 2).

**Table:** **Table 2** Summary of primary and secondary outcomes included in the study.

Outcomes	n (%)
Proportion of subjects with AE/SAE within 72 hours after hemostat application	0 (0%)
Proportion of subjects with device-related SAE within 30 days of the procedure	0 (0%)
Proportion of subject mortality within 30 days of procedure (all-cause)	0 (0%)
Proportion of subjects with acute procedural hemostasis (index endoscopy)	59 of 59 (100%)
Proportion of subjects with clinical signs of rebleeding in 72 hours post procedure, any source	3 (5.1%)
Proportion of subjects with clinical signs of rebleeding at the treatment site in 72 hours post procedure verified in second-look endoscopy	2 (3.4%)
AE, adverse event; SAE, serious adverse event.	

In addition, the study evaluated average blood glucose levels among diabetic (n = 18) and non-diabetic (n = 42) patients before and after the procedure, measured at 2 hours and 4 hours post-procedure. Pre-procedure, individuals with diabetes exhibited a mean blood glucose concentration of 149 ± 84.5 mg/dL, whereas those without diabetes had a concentration of 111 ± 12.3 mg/dL. Following the procedure, no significant elevation in blood glucose levels was observed in either diabetic or non-diabetic subjects. Specifically, diabetic patients recorded mean post-procedure blood glucose concentrations of 130.4 ± 65.8 mg/dL and 132 ± 50.6 mg/dL at 2 and 4 hours, respectively. For non-diabetic patients, the corresponding values were 111.3 ± 9.1 mg/dL and 113.9 ± 13.1 mg/dL at 2 hours and 4 hours, respectively.

## Discussion

In this prospective single-arm study, we report outcomes with the novel plant- and powder-based proprietary Resolv Endoscopic Hemostat System for treating UGIB. There was a 100% rate of achieving primary hemostasis with 5.1% rebleeding rates. In addition, no AEs related to the device or the agent were observed. This study provides evidence of new powder-based agents composed entirely of a plant-based polysaccharide with no animal proteins and no unique mixing or storage requirements. This includes accessibility to bleeding sites in challenging locations and the ability to treat a wider bleeding area.

Existing HPs in the market include Hemospray, EndoClot, and UI-EWD. 6 In a systematic review and meta-analysis of 27 studies (N = 1916) reporting outcomes of Hemospray with UGIB of various etiologies, the pooled hemostasis rate was 94.5% with a rebleeding rate of 9.9% in 3 days and 17.6% in 30 days 8 . The addition of TC-325 to conventional treatment led to a higher rate of immediate hemostasis than conventional treatment alone, with an odds ratio of 4.40 8 . Several cases of self-limited abdominal pain and viscous perforation have been reported in the literature 6 9 10 11 12 . The rate of hemostasis was 64% in patients with UGIB treated with EndoClot as a first-line therapy in a prospective study 13 . Rates of recurrent bleeding range from 11% to 23% after EndoClot therapy 13 14 15 . In a study of 154 patients comparing Hemospray and EndoClot (n = 154), the overall success rate for short-term hemostasis (72 hours), long-term hemostasis (30 days), and the incidence of recurrent bleeding were 82%, 69% and 21%, respectively, with no significant difference between both groups 16 . Efficacy of UI-EWD was reported to be 94% for immediate hemostasis with a 30-day rebleeding rate of 19% 17 . In our study comprising 59 cases, immediate and 72-hour success rates for hemostasis were notably higher, at 100% and 94.9%, respectively.

Resolv as monotherapy achieved hemostasis on the index procedure 100% of the time, with a post-procedure rebleeding rate of 5.1% (all sites) for Forrest Ib bleeding. Current literature suggests that HPs are ineffective as first-line therapy for Forrest Ia bleeds. However, utilization of HPs remains a viable option in this specific context, serving as a potential bridge or rescue strategy while awaiting implementation of an alternative therapeutic approach. When it comes to managing diffuse tumor bleeding, difficulties often persist despite traditional endoscopic treatment methods. However, studies have demonstrated that applying a hemostatic powder might offer a potential solution in these scenarios, serving as an effective definitive or interim measure before implementing radiotherapy or surgical intervention. The Resolv hemostatic powder demonstrated effectiveness in diverse cases, including bleeding in patients with chronic liver disease and portal hypertensive gastropathy/duodenopathy, as well as malignancy or peptic/duodenal ulcers. Moreover, it proves beneficial following various endoscopic procedures such as ESD, EMR, sphincterotomy, and polypectomy.

The Resolv Endoscopic Hemostat System is similar to NexStat Plus, a topical hemostatic agent applied during ear, nose, and throat surgeries to manage bleeding. It is naturally expelled from the application site through the throat and gastrointestinal tract. Notably, over 5000 NexStat Plus devices have been employed since 2015, with no reports of issues, adverse effects on patients, or recalls associated with use of NexStat Plus devices since its introduction to the market. The Resolv Endoscopic Hemostat System incorporates maltodextrin powder obtained from potato starch, ensuring safety for individuals with celiac disease and gluten intolerance. It is also safe to use in patients with lactose intolerance, unlike UI-EWD. Despite the role of maltodextrin in raising blood glucose levels, our study revealed no instances of hyperglycemia, establishing its safety for diabetic and non-diabetic patients. In our study, the Resolv Endoscopic Hemostat System was not associated with AEs. No incidents of bowel obstruction, perforation, or particulate embolization were noted.

Our initial study yielded promising results with Resolv hemostatic powder for managing UGIB, but it also had several limitations. First, the sample size was limited, and because the product has not yet been released, additional clinical studies are imperative to establish the effectiveness of the Resolv system. Second, it was a single-arm study, and the system has not been compared with standard endoscopic interventions such as hemoclips or thermal coagulation. Third, its applicability to ampullary lesions was not studied due to the associated risk of developing pancreatitis. Selection bias is also another limitation of this study. The study was conducted at a large tertiary care center, which might limit its widespread application. Despite these limitations, no severe bleeding or hemorrhage-related events were observed, and a notably higher hemostasis rate was evident in patients treated with the hemostatic powder from the Resolv system. Nevertheless, this is the first study and reported outcome of the Resolv Endoscopic Hemostat System for managing UGIB for Forrest 1b lesions and adds a tool in the endoscopist armamentarium for treating UGIB. Based on our study, Resolv has been granted clearance by the US Food and Drug Administration for management of UGIB 18 .

## Conclusions

In conclusion, the Resolv Endoscopic Hemostat System is a safe and effective device for achieving immediate hemostasis in patients with NVUGIB. Future studies are required to examine its widespread adoption and applicability.
